# NMR insights into dynamic, multivalent interactions of intrinsically disordered regions: from discrete complexes to condensates

**DOI:** 10.1042/EBC20220056

**Published:** 2022-12-16

**Authors:** Rashik Ahmed, Julie D. Forman-Kay

**Affiliations:** 1 Program in Molecular Medicine, The Hospital for Sick Children, Toronto, ON M5G 0A4, Canada; 2 Department of Biochemistry, University of Toronto, Toronto, ON M5S 1A8, Canada

**Keywords:** biomolecular condensates, fuzzy complexes, intrinsically disordered proteins, NMR spectroscopy, phase separation

## Abstract

The spatial and temporal organization of interactions between proteins underlie the regulation of most cellular processes. The requirement for such interactions to be specific predisposes a view that protein–protein interactions are relatively static and are formed through the stable complementarity of the interacting partners. A growing body of reports indicate, however, that many interactions lead to fuzzy complexes with an ensemble of conformations in dynamic exchange accounting for the observed binding. Here, we discuss how NMR has facilitated the characterization of these discrete, dynamic complexes and how such characterization has aided the understanding of dynamic, condensed phases of phase-separating proteins with exchanging multivalent interactions.

## Introduction

Protein–protein interactions underlie the execution and regulation of most cellular processes. The fidelity and specificity with which these protein–protein interactions must occur to facilitate controlled changes in cellular processes were initially explained by stable structure within interfaces and complexes between binding partners [1]. However, it has become increasingly clear that many cellular processes are governed by complexes involving intrinsically disordered proteins/regions (IDPs/IDRs), which lack stable secondary and tertiary structure [2]. These findings have challenged the paradigm that stable interfaces are a universal requirement for protein interactions [3]. This discrepancy was partially reconciled by observations of IDPs/IDRs assuming different folded structures upon binding to various targets through coupled binding and folding [4]. Moreover, the finding that disordered proteins can transiently sample folded structures that function as pre-formed molecular recognition elements (MoRFs) for their cognate binding partners reinforced the notion that stable structure underlies binding [4].

Nevertheless, bioinformatic analyses predict that conditionally folded IDRs, such as those that fold upon binding, account for only ∼20% of the disordered human proteome [5]. Rather, many interactions lead to ‘fuzzy complexes’ with disordered character remaining in the bound state [6]. Indeed, an increasing number of complexes involving IDPs/IDRs show retention of disorder in the bound state with minimal or transient ordering around sites of binding [7–13]. In such complexes, an ensemble of conformations in dynamic exchange account for the observed binding, as opposed to a single bound conformation [7]. In the case of IDRs interacting with folded partners, binding is often mediated by short contiguous stretches of disorder (∼3–11 residues) known as short linear interacting motifs (SLiMs), which act as interaction modules [14,15]. SLiMs usually have relatively low affinities (*K*_D_ ≥ μM) for their binding partners, affording interactions that are both transient and reversible, making them ideal for mediating dynamic processes such as cell signalling. Notably, while each SLiM may bind weakly to the interacting partner, the presence of multiple SLiMs or multiple cognate folded partner domains increases the overall valency and consequently affinity of the interaction (*K*_D_ < μM) [15]. Such higher affinity, dynamic interactions have also been reported for complexes formed by two IDRs [11]. Thus, dynamic or fuzzy complexes expand the narrow definition of binding, from requiring stable structure to utilizing multivalent, dynamic interactions to mediate biological function.

IDRs adopt a large ensemble of rapidly interconverting conformations in solution owing to their shallow free energy landscape [2,16]. The shallow energy landscape of IDPs makes them remarkably poised to respond to small solution perturbations, allowing for context-dependent changes in IDR-driven biological regulation. Moreover, such structural plasticity offers several advantages over conventional folded domains. These include conferring accessibility to enzymes that incorporate post-translational modifications [17] and binding to several target molecules with similar affinity [18], i.e., multispecificity. As well, plasticity facilitates the formation of flexible scaffolds for assembly of large networks of biomolecules, such as cellular condensates [19]. Yet, widescale appreciation of the biological role of highly dynamic and disordered protein complexes has been slow. This is partially attributed to the dramatic progress in X-ray crystallography and cryo-electron microscopy (cryo-EM), which has enabled rapid structural characterization of large numbers of protein complexes [20] and reinforced the view that protein–protein interactions are relatively static and are formed through unique and stable complementarity of the interacting partners.

A compelling shift in this view, however, was catalyzed by developments in nuclear magnetic resonance spectroscopy (NMR) [21–25], which have provided atomic-resolution insights on protein dynamics – a critical element largely missing from the snapshots generated through crystallography and cryo-EM. Here, we discuss the development of key concepts related to dynamic, multivalent interactions of IDRs, using primarily examples from our own work, and highlight the critical role of NMR in elucidating the dynamics within these complexes. We also describe how the characterization of multivalent interactions and discrete complexes has aided the understanding of dynamic large-scale associated states, e.g., condensed phases of phase-separating proteins, with exchanging multivalent interactions.

### Discrete dynamic multivalent complexes

In the early 2000s, a closer examination of the structures of many complexes revealed that the regions of proteins that contribute productively to binding often cannot be described by a single bound conformation, leading to their description as ‘fuzzy complexes’ [7]. In such complexes, one or more of the interacting partners remain dynamic – either by adopting multiple alternate conformations, having disordered segments that either flank or connect ordered binding regions, or in the extreme scenario remain completely disordered. While many examples of polymorphic or partially ordered complexes were demonstrated, dynamic, disordered complexes were, for the most part, poorly characterized, with some data for elastin, a large-scale associated homo-oligomerized state of tropoelastin monomers [8].

#### Characterization of discrete dynamic multivalent complexes – spectroscopic challenges and solutions

Characterization of such putative dynamic complexes proved challenging given that conformational heterogeneity of disordered regions makes alignment of 2D projections in cryo-EM single particle analysis challenging [26] and hinders protein self-organization into a translationally periodic arrangement for successful crystallization [27]. In cases where conformational heterogeneity is sufficiently reduced, through addition of suitable ligands, inhibitors or cofactors, X-ray crystallography and cryo-EM provide only a static view of the complex locked in a single conformation. In addition, techniques used to quantify the strength of biomolecular interactions, e.g., isothermal calorimetry (ITC), surface plasmon resonance (SPR), etc., only provide a measure of the global binding and do not delineate the individual contributions of local binding motifs.

In contrast, NMR spectroscopy provides an ensemble-averaged, atomic-resolution view of the interacting proteins and is thus sensitive to each of the bound conformers that comprise the dynamic complex [28]. Moreover, NMR spin-relaxation and magnetization-exchange experiments are sensitive to dynamic processes spanning timescales ranging from picoseconds to seconds, providing insight into both local (side chain rotations and loop motions) and global events (domain reconfigurations, and folding/unfolding) [25]. Hence, NMR is ideally suited for the characterization of dynamic protein complexes involving disordered protein.

#### Sic1:Cdc4 – Early example of discrete dynamic multivalent complex

**Figure 1:**
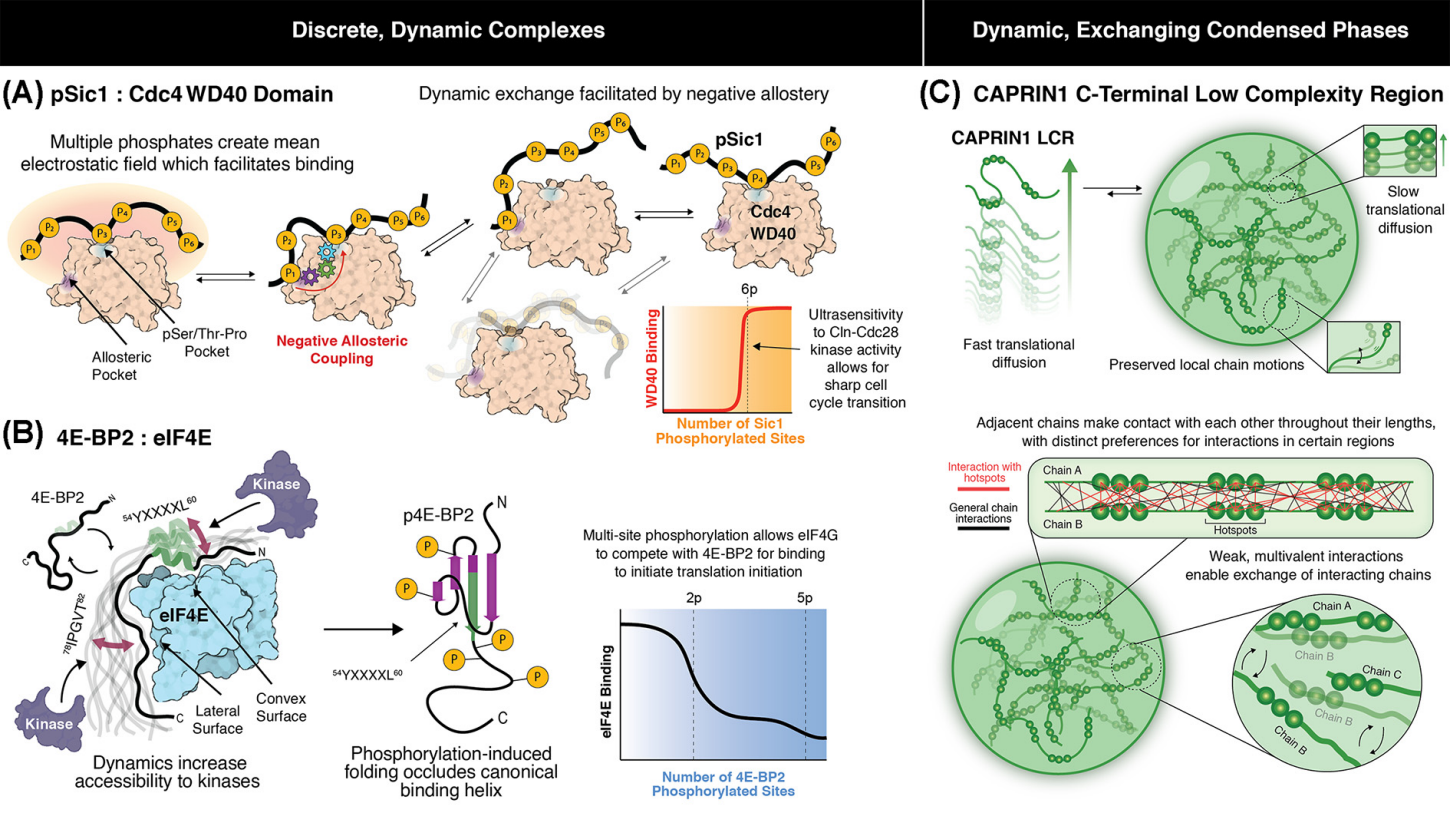
Schematic highlighting key principles governing discrete, dynamic complexes of IDRs and dynamic, condensed phases of IDRs with exchanging interactions (**A**) Biophysical model for the discrete, dynamic complex formed by Sic1 and the WD40 domain of Cdc4. Multisite phosphorylation of Sic1 (yellow circles) creates numerous weak binding motifs termed Cdc4 phospho-degrons (CPDs) that synergize to yield higher affinity interactions with the positively charged pSer/Thr-Pro pocket (blue-shaded region) of Cdc4. Disorder is retained in the bound state, with transient ordering only around sites of binding. Multiple phosphorylation sites create a mean negative electrostatic field that facilitates retention of pSic1 in the vicinity of Cdc4 (left). Binding of CPDs to the pSer/Thr-Pro binding pocket tethers additional CPDs in close proximity to the weaker affinity allosteric site (purple-shaded region). Negative coupling between the allosteric site and the pSer/Thr-Pro pocket disengages bound CPDs at the pSer/Thr-Pro pocket for subsequent rebinding or dynamic exchange with alternate CPDs (right). Multisite phosphorylation sets a threshold for recognition and elimination of Sic1, leading to precise transition between cell-cycle phases (lower right). (**B**) Model for the dynamic, bipartite interaction of eIF4E with 4E-BP2. The canonical (^54^YXXXXL^60^) and secondary (^78^IPGVT^82^) eIF4E-recognition motifs of 4E-BP2 bind to the convex and lateral surfaces of eIF4E, respectively, resulting in the formation of a bipartite interface (left). Significant structural plasticity is retained in the eIF4E-bound state of 4E-BP2, resulting in sampling of heterogeneous conformations (left). On-off exchange dynamics of 4E-BP2 increase accessibility to kinases (left). Phosphorylation induces folding of 4E-BP2 into a 4-stranded β-domain with residues of the canonical binding helix buried in the interior (middle), allowing eIF4G to compete with 4E-BP2 to trigger translation initiation (right). (**C**) Biophysical model of the intra- and inter-chain dynamics and interactions within the condensed phase of CAPRIN1 C-terminal IDR. Translational diffusion is significantly attenuated in condensed phases of CAPRIN1 LCR, while local chain motions are preserved (top). Local motions facilitate transient contacts between IDR chains with distinct preferences for interactions in certain “hotspot” regions (bottom). The weak, multivalent nature of the interactions allow chains to disengage and reconfigure into different poses that bring various segments of the chains in close proximity as well as enable exchange of interacting chains.

One of the earliest characterizations with residue-specific biophysical approaches of a discrete, dynamic complex involving disordered protein was for the interaction between the disordered yeast cyclin-dependent kinase (CDK) inhibitor Sic1 and its cognate binding partner, the F-box protein Cdc4 [13]. Phosphorylation of Sic1 by Cln-Cdc28 kinase directs Sic1 to the SCF^Cdc4^ ubiquitin ligase, resulting in ubiquitination and degradation in late G1 phase and subsequent transition into S phase [29,30]. The WD40 domain of Cdc4 recognizes phosphorylated serine/threonine sequences in Sic1, termed Cdc4 phosphodegrons (CPDs), primarily through a single pSer/Thr-Pro binding pocket [31,32]. Multiple suboptimal CPDs exist in Sic1 and binding of Sic1 to Cdc4 with biologically relevant affinity (*K*_D_ ∼ 1 μM) requires multi-site phosphorylation [33], suggestive of a binding mode wherein multiple CPDs bind to a single site in Cdc4 in dynamic equilibrium (Figure 1A).

#### NMR characterization of Sic1:Cdc4 discrete dynamic multivalent complex

NMR was exploited to characterize the binding mode of the multiple phosphorylation sites in pSic1 to Cdc4. For a protonated, stable ∼80 kDa complex, line broadening due to rapid transverse relaxation would preclude the detection of NMR signal. Yet, ^1^H^N^ – ^15^N correlation NMR spectra recorded for ^15^N-labeled pSic1, in the presence of excess Cdc4 at concentrations at which ∼99% pSic1 is bound, displayed many observable resonances [13]. This observation indicates that pSic1 does not form a stable structure in the context of its complex with Cdc4 but rather is undergoing dynamic exchange between states with elements that are tightly engaged with Cdc4 and those that are not engaged. Interestingly, the observable pSic1 signals exhibited limited amide proton chemical shift dispersion, characteristic of a disordered protein for which fast interconversion of different conformers lacking significant secondary and tertiary contacts produces an averaged magnetic environment. Taken together, these findings indicated that pSic1 remains largely disordered in complex with Cdc4 and exhibits significant on–off exchange dynamics (Figure 1A).

Further insight into the exchange dynamics was gathered from transferred cross-saturation (TCS) NMR experiments [34]. In this experiment, the aliphatic protons of protonated Cdc4 are saturated and the saturation transfer to amide protonated, aliphatic deuterated pSic1 (26:1 molar ratio of pSic1:Cdc4) is detected through a ^1^H^N^ – ^15^N correlation readout of the free pSic1 signal. Notably, given the large excess of pSic1 in the sample, only minimal (∼4%) transferred cross-saturation is expected. However, significant (and variable) TCS effects beyond the expected values were mapped to the multiple CPDs in pSic1. This observation not only indicates binding of such sites to Cdc4 but also suggests that the various CPDs have locally distinct affinities leading to residue-specific buildup of saturation transfer rather than a simple two-state on–off equilibrium [13]. This finding is consistent with the multiple CPDs rapidly exchanging on and off of the Cdc4 interaction surface in dynamic equilibrium within the context of the ‘bound’ state. In support of this, a mean-field statistical model of the complex [35] predicts the pSic1–Cdc4 interactions to be highly transient, relying not only on more specific short-range contacts with the binding pocket but also on less specific long-range polyelectrostatic interactions between the binding partners.

Despite the transient nature of individually weak CPD contacts within this interaction, pSic1 binds to Cdc4 with *K*_D_ ∼ 1 µM [33]. Synergistic binding of multiple CPDs is in part mediated by compaction of the disordered chain, which is stabilized by long-range tertiary contacts in pSic1, as detected through paramagnetic relaxation enhancement (PRE) and pulsed-field gradient (PFG) diffusion NMR experiments [13]. Compactness could increase the spatial density of negative charge and generate an average negative electrostatic field that draws pSic1 into the positively charged Cdc4 binding surface. Consequently, other CPDs that are tethered in close proximity to Cdc4 can bind, either through exchange of CPDs at the canonical pSer/Thr-Pro binding pocket or engagement at a distal allosteric site (Figure 1A). Notably, the allosteric pocket in Cdc4 plays a critical role in facilitating the dynamic exchange of pSic1 CPDs [36], adding yet another layer of regulation of this dynamic, disordered complex.

The distal basic allosteric pocket in Cdc4 can simultaneously engage a second phosphorylated residue in pSic1 (Figure 1A) [37]. To identify the role of the allosteric pocket, the isolated WD40 domain of Cdc4 was interrogated in the free state and in complex with various phosphorylated Sic1 peptides [36]. Given the large size of the WD40 domain of Cdc4 (40 kDa), methyl-TROSY NMR experiments, which exploit the favorable relaxation properties and sensitivity of protonated methyl resonances in otherwise deuterated samples, were employed [39]. Methyl cross-saturation experiments, analogous to the amide-detected TCS experiments described above, showed significant attenuation of methyl resonances at both the canonical and allosteric pockets upon addition of extended multisite phosphorylated Sic1 (pSic1 and a 20 residue phosphorylated peptide Sic1^20pS69/pS76/pS80^), confirming binding at both sites [36]. In contrast, binding at the allosteric site was not observed upon addition of short phosphopeptides (Sic1^10pT2/pT5^, Sic1^9pT45^, Sic1^9pT173^), suggesting that tethering of pSic1 at the primary CPD pocket enables binding at the lower-affinity allosteric site. Unexpectedly, perturbing mutations introduced at the allosteric pocket enhanced binding of Sic1^20pS69/pS76/pS80^ to Cdc4, consistent with negative allosteric coupling between the allosteric site and the primary pocket. Reinforcing this, phosphorylated peptides sufficiently long to bind both sites, e.g., Sic1^20pS69/pS76/pS80^, showed reduced chemical shift perturbations at the primary site, compared to shorter peptides, e.g., cyclin E, which only bind at the primary site.

Taken together, a biophysical model for dynamic site exchange emerged wherein binding of one CPD to the primary pocket tethers other CPDs in close proximity to the Cdc4 surface, thereby enhancing interactions with the lower-affinity allosteric site [36]. CPD binding to the allosteric site weakens interactions at the primary pocket, facilitating the dissociation and subsequent exchange of CPDs at the primary pocket (Figure 1A). Importantly, binding at the allosteric site provides a physical basis for tethering of pSic1 to Cdc4 upon transient disengagement of the primary pocket, a mechanism which works in concert with the local negative electrostatic field generated by the collection of negatively charged phosphates on Sic1. Thus, there is no single unique binding interface for pSic1:Cdc4, and the resulting complex remains significantly dynamic. This mode of binding strongly diverges from views of protein-protein interactions based on stable interfaces within a uniquely defined stable complex [40].

#### Functional importance of dynamic, multivalent interactions of Sic1:Cdc4

Notably, dynamic site exchange of Sic1 is critical for proper cell-cycle regulation. Mutation of Sic1 to contain a single phosphorylated optimal CPD site targeting the primary pocket results in yeast cell death due to premature cell cycle transition [33]. In contrast, multisite phosphorylation of suboptimal CPDs sets a threshold for recognition and subsequent elimination of Sic1 that is coincident with a graded increase in Cln-Cdc28 kinase activity [33,38]. Such a sharp, switch-like response, i.e., ultrasensitivity to Cln-Cdc28, affords rapid and regulated cell-cycle transition (Figure 1A). Importantly, the on-off exchange dynamics of pSic1 and the retention of disorder in the bound state increases the accessibility of the binding interface to modifying enzymes, such as kinases and phosphatases, which fine-tunes the binding as required by the cell. Thus, dynamic interactions within IDR-containing complexes play important functional roles in biology, as exemplified here for pSic1:Cdc4.

#### Other notable examples of discrete dynamic multivalent complexes

Similar dynamic, polyvalent electrostatic interactions have also been reported for the interactions between two IDRs, the highly positively charged (+53) linker histone H1.0 (H1) and the highly negatively charged (-44) prothymosin α (ProTα) [11]. Limited amide proton chemical shift dispersion and NMR secondary chemical shift analyses indicated the absence of pronounced tertiary structure for ProTα when bound to H1. Intramolecular distance maps derived from NMR, smFRET and simulations suggested an interaction surface that is broadly distributed across the sequences of the two proteins, mapping in accordance with the distribution of complementary charged surfaces. Notably, the simulated structural ensemble did not identify unique clusters of bound conformations, suggestive of the absence of defined binding sites or persistent interactions between specific residues [11]. Collectively, the NMR-based measurements discussed here provide evidence that dynamic complexes involving IDRs contain weaker, multivalent interaction sites that synergize to yield higher affinity interactions analogous to those demonstrated for interactions defined by stable protein interfaces.

### Discrete dynamic complexes

#### 4E-BP2:eIF4E – High affinity discrete dynamic bipartite complex

While high affinity interactions are generally ascribed to stable complexes formed between folded domains or a folded domain and a SLiM, a growing body of evidence indicates that tight binding can also occur for complexes involving IDPs/IDRs that retain significant dynamics in the bound state. A notable example is the complex formed by eukaryotic initiation factor 4E (eIF4E) and the disordered eIF4E binding protein 2 (4E-BP2), which interact with nM affinity (*K*_D_ = 3.2 ± 0.6 nM) [41]. eIF4E binds the 7-methylguanosine-containing cap of mRNA and recruits it to the eIF4F-ribosome complex through association with the scaffolding protein eIF4G [42]. 4E-BPs and eIF4G share a common eIF4E binding motif (YXXXXL) and occupy an overlapping surface on eIF4E. Thus, binding of 4E-BPs to eIF4E sterically blocks binding to eIF4G and inhibits assembly of the translation initiation complex [42]. NMR characterization of the 4E-BP2:eIF4E complex revealed a second 4E-BP2 segment, in addition to the canonical helix formed by the YXXXXL binding motif, that interacts with eIF4E [41]. A crystal structure of a 4E-BP1 peptide fragment in complex with eIF4E shows that the canonical and secondary sites bind to the convex and lateral surfaces of eIF4E, respectively, resulting in the formation of a bipartite interface (Figure 1B) [43]. However, unlike the stable complex suggested by the crystal structure, NMR investigations using full-length 4E-BP2 revealed a more dynamic mode of binding [41]. This example highlights the utility of NMR, which specializes in probing dynamic states that are not amenable to structural determination by crystallography.

#### NMR characterization of 4E-BP2:eIF4E dynamic complex

Indeed, addition of eIF4E to full-length 4E-BP2 results in NMR signal broadening of 4E-BP2 resonances without migration of peaks or appearance of bound state resonances [41]. These observations are consistent with slow exchange (on the NMR timescale) between free and bound states, as expected for a high-affinity (nM *K*_D_) interaction, with µs – ms conformational exchange within the complex broadening bound resonances beyond detection. Such conformational exchange can be interpreted as the two segments of the bipartite interface coming on and off the surface of eIF4E, resulting in sampling of heterogeneous conformations within the bound state, i.e., a fuzzy complex (Figure 1B). To test the hypothesis that 4E-BP2 binds eIF4E through such a dynamic, bipartite interface, perturbing mutations were introduced at both canonical and secondary sites to weaken binding and minimize contributions from conformational fluctuations within the complex. In conjunction, NMR spectra were recorded at increasing temperatures to modulate the chemical exchange regime, leading to observation of bound state resonances [41]. Notably, the two interacting segments of the bipartite interface contribute synergistically to binding as the isolated canonical site peptide binds in the µM range and no significant binding is detected for a peptide containing only the secondary site [44]. Therefore, NMR interrogation of the 4E-BP2:eIF4E complex demonstrated that high affinity interactions can be mediated by the collective contributions of lower-affinity segments which retain significant structural plasticity in the bound state (Figure 1B).

#### Functional importance of dynamic interactions of 4E-BP2:eIF4E

Such structural plasticity is critical to regulation of cap-dependent translation, since release of the translational inhibitor 4E-BP2 from eIF4E is coupled to multisite phosphorylation of 4E-BP2 [42]. Unlike stable high-affinity complexes which have slow dissociation kinetics, the exchange dynamics of 4E-BP2 within the complex with eIF4E facilitates the transient exposure of phosphorylation sites for modification by kinases (Figure 1B). Dynamic interactions therefore enable formation of ternary complexes, to facilitate regulation without the need for complete release of binding partners. Docking of kinases to 4E-BP2:eIF4E leads to multisite phosphorylation [45]. This, in turn, induces folding of 4E-BP2 into a 4-stranded β domain which occludes the YXXXXL binding motif [46] (Figure 1B), allowing eIF4G to compete with 4E-BP2 for binding to initiate translation.

#### Other notable examples of discrete dynamic complexes

Regulatory switch-like behavior was also reported for the ternary complex formed by HIF-1α, CITED2 and the TAZ1 domain of the transcriptional coactivators CREB-binding protein (CBP) and p300 [47–49]. Under conditions of oxygen deprivation, the transcription factor HIF-1α binds the TAZ1 domain of CBP and p300 to induce transcription of adaptive genes, including the negative regulator CITED2 which directly competes with HIF-1α for binding to TAZ1. NMR, mutagenesis and kinetic studies suggest that HIF-1α remains highly dynamic in complex with TAZ1 allowing for initial binding of CITED2 to a partially overlapping site on TAZ1 without requiring dissociation of HIF-1α [48]. Subsequent engagement of other CITED2 binding elements results in a unidirectional, switch-like release of HIF-1α from the complex. Taken together, these findings suggest that dynamic interactions involving multiple IDR binding elements encode unique, sharp binding transitions that facilitate precise regulation of key biochemical processes.

Another intriguing example of an IDR engaging in dynamic complexes is found in the Cystic Fibrosis Transmembrane Conductance Receptor (CFTR) regulatory (R) region, which acts as a dynamic exchanging hub that integrates numerous phospho- and calcium-dependent intra- and intermolecular interactions [50,51]. Collectively, the findings established for these and other discrete, dynamic complexes [52–55] have several general implications. First, weak binding motifs in IDRs have the potential to interact with several target molecules with similar affinity, i.e., multispecificity. Second, the retention of disorder in the bound complex can confer flexibility to the chain which may facilitate scaffolding of larger complexes. Lastly, the structural plasticity and associated dynamics of IDRs in the bound state renders the protein chain accessible to post-translational modifications, which can fine-tune the strength of the interacting elements and enable rapid exchange of binding partners. In the following section, we describe how the principles gathered from studies of discrete, dynamic complexes provide a foundation for understanding interactions within dynamic, large-scale associated states, such as condensed phases of phase-separating proteins with exchanging multivalent interactions.

### Dynamic exchanging multivalent interactions in biomolecular condensates

#### Principles governing discrete, dynamic complexes provide foundation for understanding condensed phases

Synergistic multivalent interactions between interacting biomolecules are fundamental to the formation of biomolecular condensates via phase separation and other related physical processes [56,57]. IDRs often play a critical role, either by directly binding to interacting partners, i.e., containing SLiMs or chemical group ‘stickers’, or indirectly by conferring chain flexibility or modulating chain solubility, i.e., serving as ‘spacers’, in the stickers and spacers model [58,59]. The resulting network of interactions leads to macroscopic assemblies that span length-scales far larger than the discrete complexes involving IDRs described above. Yet, the principles governing the assembly and regulation of these mesoscopic interacting networks in condensed phases of phase-separating systems build on those described for the discrete, dynamic complexes of IDRs.

Indeed, NMR characterization of macroscopic condensed phases formed by IDRs of RNA-binding proteins, e.g., cell cycle associated protein 1 (CAPRIN1), Fragile-X mental retardation protein (FMRP) and deadbox helicase 4 (DDX4), indicate that IDRs retain disorder and significant local dynamics in the condensed phase despite a reduction in translational diffusion by over two orders of magnitude [24,60–63] (Figure 1C). Notably, such dynamics afford IDRs the capacity to participate in weak, multivalent interactions that rapidly form and break and exchange with alternate sites either on the same chain or alternate chains (Figure 1C), analogous to those observed between pSic1 and Cdc4, albeit significantly weaker. The dynamic exchange of interacting sites facilitates the incorporation of a large number of protein molecules into the network, resulting in a large-scale associated condensed phase. Such dynamic interactions could also be heterotypic, arising from interactions between different proteins or proteins and nucleic acids [64]. While computational simulations [65–68] and theoretical approaches [69] have provided detailed descriptions of the behavior of phase-separating systems, recapitulating many experimentally observed trends and correctly predicting complex multiphase behaviours [67], validation of these predictions is required using experimentally determined site-specific information.

#### NMR characterization of CAPRIN1 condensed state

In principle, NMR can provide atomic resolution insight on highly dynamic systems such as those in condensed protein states. Indeed, ^1^H-^1^H nuclear Overhauser effects (NOEs), which report on short-range contacts between pairs of interacting protons, can be exploited to generate maps of the inter-molecular contacts that drive phase separation [70]. To this end, sensitive 2D NMR experiments were developed wherein proton spins are excited in an amino acid-type manner (Arginine/Glycine/Serine/Aromatics) or more generally (non-glycine residues) and magnetization is transferred from such spins to backbone amides via the NOE and quantified through a ^1^H-^15^N correlation readout [62]. Accordingly, contacts between H^C^ protons of specific amino acid types and backbone amides (H^N^) of individual residues can be mapped, either on the same chain (intramolecular) or adjacent chains (intermolecular). A sample comprised of ^12^C, ^15^N, ^2^H and ^13^C, ^14^N, ^1^H-labeled CAPRIN1 IDR can be used to filter out intra-molecular contacts and enable mapping inter-molecular contacts of CAPRIN1 in the condensed phase [62].

Intermolecular NOE maps derived for the C-terminal low complexity region of CAPRIN1 using this approach identify strong connectivities throughout the sequence, with significantly higher values centered around aromatic- and arginine-containing regions spanning G624-R626, G638-R640 and R660-Q666 [62]. Connectivities between side-chain aromatic protons and backbone amide protons of glycines, arginines and glutamines are pervasive [62]. Considerably stronger NOEs arise from tyrosine compared to phenylalanine sidechains, consistent with greater significance of tyrosines versus phenylalanines in phase separation [71]. For certain residues, whose methyl resonances are sufficiently well-resolved in the ^13^C dimension, site-specific information can be gathered for both the origination and destination of magnetization, e.g., between Hδ2 of L621 in one chain and the amide proton of V708 in a neighbouring CAPRIN1 [62]. These findings highlight the underappreciated role of backbone interactions in phase separation which may be stabilized through amide hydrogen bonding and/or π interactions [62]. Collectively, such intermolecular contact maps can be envisioned as adjacent CAPRIN1 chains weakly associating at interaction hotspots, which further break and reconfigure into different poses that bring various segments of the chains in close proximity (Figure 1C). In support of the importance of these interaction hotspots, Ala-Ser-Ala mutations introduced at hotspot sites and O-GlcNAc modification of S644 and S649 significantly decrease CAPRIN1 phase separation [62].

#### Post-translational modifications alter protein chain biophysics and phase separation propensity

Post-translational modifications of IDRs regulate the formation and dissolution of condensed phases by modulating the biophysical characteristics and specific interactions of protein chains [59,72–74]. Multisite phosphorylation of the low-complexity C-terminal region of FMRP (FMRP_LCR_) increases the negative charge near glutamic/aspartic acid (E/D) clusters and consequently their potential to form multivalent electrostatic interactions with positively charged arginine/lysine (R/K) clusters [75]. Accordingly, phosphorylated FMRP (pFMRP_LCR_) readily undergoes phase separation at concentrations well below that required for non-phosphorylated FMRP_LCR_. Moreover, phosphorylation enhances heterotypic interactions between FMRP and CAPRIN1, enabling co-phase separation of the two IDRs [63]. NMR characterization of the FMRP-CAPRIN1 interactions identify two arginine-rich regions of CAPRIN1 that undergo greater intensity losses in the presence of pFMRP *vs*. FMRP, suggestive of more pronounced interactions between the phosphates of pFMRP and CAPRIN1 arginine residues [63]. Thus, the accessibility of IDR primary sequences to modifying enzymes enables the introduction of a large repertoire of cellular modifications that regulate phase separation, which can be probed via NMR.

## Concluding remarks

Dynamic, sometimes disordered, complexes confer unique advantages that stable interfaces lack, including enabling switch-like binding transitions, i.e., ultrasensitivity, increasing accessibility to enzymes for PTM-induced regulation, facilitating exchange of competing partners, as well as allowing formation of discrete hubs and large networks such as in biomolecular condensates. In many cases, dynamic interactions have been demonstrated to be critical for proper cellular function underscoring the importance of studying these dynamics states beyond stable structure. There remains a significant challenge in characterizing these ensembles which do not ‘sit still’ or yield a small number of states to facilitate crystallography or cryo-EM analysis. NMR has enabled characterization of the ensembles that represent these dynamic complexes, contributing to an expanded understanding of the nature of protein interactions. NMR is now poised, in concert with other solution data such as from SAXS and single molecule fluorescence and with computational tools [76], to provide detailed atomic-level descriptions of these ensembles to illuminate the physical basis of these functional, dynamic complexes.

## Summary

- Weak, multivalent interactions of IDRs synergize to yield higher affinity interactions with fast on-off exchange dynamics not typically afforded by folded domains.
- IDRs can exhibit significant conformational fluctuations in complex with interacting partners.
- IDR dynamics in the bound state render the protein chain accessible to post-translational modifications, which can fine-tune the strength of the interacting elements and enable rapid exchange of binding partners.
- Exchange of multivalent IDR elements within discrete dynamic complexes is analogous to that within condensed phases of phase-separating proteins.
- NMR spectroscopy is uniquely able to characterize the highly dynamic exchange within discrete complexes and condensed phases involving IDRs.

## Abbreviations

4E-BP1: eukaryotic translation initiation factor 4E binding protein 1
4E-BP2: eukaryotic translation initiation factor 4E binding protein 2
CAPRIN1: cell cycle associated protein 1
CBP: CREB-binding protein
CDK: cyclin-dependent kinase
CFTR: cystic fibrosis transmembrane conductance receptor
CITED2: CBP/p300 interacting transactivator with Glu/Asp rich carboxy-terminal domain 2
CPD: Cdc4 phosphodegron
Cryo-EM: cryo-electron microscopy
DDX4: DEADbox helicase 4
eIF4E: eukaryotic translation initiation factor 4E
eIF4F: eukaryotic translation initiation factor 4F
eIF4G: eukaryotic translation initiation factor 4G
FMRP: fragile-X mental retardation protein
H1: histone H1.0
HIF-1α: hypoxia inducible factor 1α
IDP: intrinsically disordered protein
IDR: intrinsically disordered region
ITC: isothermal calorimetry
LCR: low complexity region
MoRF: molecular recognition feature
NMR: nuclear magnetic resonance
NOE: nuclear Overhauser effect
PFG: pulsed-field gradient
PRE: paramagnetic relaxation enhancement
ProTα: prothymosin α
PTM: post-translational modification
SLiM: short linear interacting motif
smFRET: single-molecule fluorescence resonance energy transfer
SPR: surface plasmon resonance
TCS: transferred cross-saturation
TROSY: transverse relaxation optimized spectroscopy
